# Management of Late Onset Perthes: Evaluation of Distraction by External Fixator—5-Year Follow-Up

**DOI:** 10.1155/2014/135236

**Published:** 2014-12-14

**Authors:** Ajai Singh, Rajeshwar N. Srivastava, Prashant Shukla, Amit Pushkar, Sabir Ali

**Affiliations:** Department of Orthopaedics, King George's Medical University, Lucknow 226018, India

## Abstract

*Background*. Hip distraction in Perthes' disease unloads the joint, which negates the harmful effect of the stresses on the articular surfaces, which may promote the sound healing of the area of necrosis. We have examined the effect of arthrodiastasis on the preservation of the femoral head in older children with Perthes' disease. *Methods and Materials*. Twelve children with age more than 8 years with Perthes' disease of less than one year were treated with hip distraction by a hinged monolateral external fixator. *Observation and Results*. Mean duration of distraction was 13.9 days. These children were evaluated by clinicoradiological parameters for a mean period of 32.4 months. There was a significant improvement in the range of movements and mean epiphyseal index, but the change in the percentage of uncovered head femur was insignificant. There was significant improvement in Harris Hip score. *Conclusions*. Hip distraction by hinged monolateral external fixator seems to be a valid treatment option in cases with Perthes' disease in the selected group of patients, where poor results are expected from conventional treatment.

## 1. Introduction

Legg-Calve-Perthes' disease is characterized by idiopathic aseptic avascular osteonecrosis of the capital femoral epiphysis [1]. The age of diagnosis of this disease is usually 4 to 12 years, with an average of 6 years. The course and prognosis of Perthes' disease are difficult to predict. The prognosis of the disease depends on bone age at presentation, the sphericity of femoral head and congruency at skeletal maturity, height of the lateral pillar of the capital epiphysis at the presentation, and range of motion of hip joint [2]. In our clinical setup many patients present to us in a later stage of disease when there is already hinge abduction or poor range of movements of hip. At worst, this condition can lead to degenerative osteoarthritis during early adulthood in about half of these patients. Late onset Perthes' disease is defined as a disorder that develops after the age of 8 years [3]. This group constitutes about 20% of cases and is known for its aggressive course and poor outcome with chronic hip pain and stiffness [3]. It is widely accepted that those most at risk of a poor outcome are who develop the disease late [4]. The possible explanations to this fact are that acetabulum is unable to accommodate the congruity of deformed femoral head due to decreased elasticity of acetabulum [5] and possibly these children have less time remaining for growth and remodeling of head of femur [6]. The way to treat late onset Perthes' disease is widely controversial. The main principles of treatment have traditionally been the relief of loading and containment of head of femur [7]. The various surgical treatment options have been proposed for this late onset disease but they have some inherent drawbacks. The varus realignment femoral osteotomy may even increase the incongruity of hip as well as shortening of the limb [8]. The valgus femoral osteotomy may succeed in unloading the deformed head, but it may increase the lateral subluxation of head of femur [9]. Either of these procedures does not influence the basic avascular process of head of femur. The acetabular lateral shelf procedure [10], Salter osteotomy [2], Chiari osteotomy [2], and triple osteotomy [11] are aimed at reorienting or increasing size of acetabulum and at producing more support to head of femur. However, these approaches neither reduce the pressure on the head of femur nor change the shape of the femoral head. No traditional treatment modality has shown any statistically significant efficacy in improving the outcome of Perthes' disease.

The hip distraction with or without soft tissue release by either hinged monolateral external fixator or Ilizarov is a relatively newer modality of treatment of late onset Perthes' disease [7]. The concept of this modality of treatment is that, by creating the gap in the joint and thus decreasing the stress on the articulating surface and by maintaining some of the movements of the joint, the synovial circulation will improve. This will in fact encourage the fibrous repair of defects of the articular cartilage and further encourage the preservation of relatively intact and the congruent head of femur [12]. We present our preliminary results of this prospective trial with the aim to assess the effect of hip diastasis in a selected group of patients of late onset Perthes' disease.

## 2. Methods and Materials

Ethical permission for this study was obtained from departmental review committee. Informed consent was taken from the guardians of the children before participation in the study. Irrespective of collapse of head of femur, all children of both sexes with age of 8 years and above with Perthes' disease of less than one year of duration were included in this prospective study. The exclusion criteria included children not fulfilling the above inclusion criterion, the children suggestive of other causes of avascular necrosis such as sickle cell anaemia or multiple epiphyseal dysplasia, children who were known to be immunocompromised, children on long term steroid, children previously partly treated for this disease, and children with any history suggestive of Perthes' disease in contralateral hip or not willing to undertake this particular modality of management. To date, no child was excluded as per these exclusion criteria.

### 2.1. Operative Procedure

All children were operated on under general or regional anesthesia. Axis of hip joint was identified under image intensifier. A hinged monolateral external fixator was then applied. On the table, hip was distracted until widening of the joint was seen under image intensifier. With fixator on, the flexion-extension range was checked under anaesthesia. As soon as pain permits in the postoperative period, children were allowed for supported walking with toe touching. Within pain limits, early hip distraction started and active flexion-extension movements of affected hip were allowed. Distraction was continuous till Shenton's arch was continued or till distraction caused pain due to resistance of contracted soft tissues. Till the completion of distraction, all patients remained hospitalized. The soft tissue release or tenotomy was not performed in any of the cases as it was not required in any of our patients. At the time of discharge, attendants were told to take off loosening of frame and pin tracts. Assembly was continued till the end of 4 months (in principle, before its removal, any fixture is made to decrease the chances of stress fractures). After the assembly removal, all children were manipulated under anaesthesia and then all children were maintained on hip abduction braces with support for the next 6 months. After the external fixator removal, braces were given to avoid any stress fractures and to encourage these patients to bear weight gradually (and aim of this brace was not to modify the progression otherwise). These were used only while weight bearing. This standard protocol was uniformly practiced in all cases.

The primary outcome measures were clinical (Trendelenburg gait, range of movements, and limb length discrepancy) and radiological [13] (epiphyseal index and uncoverage percentage) (using Reimer's subluxation index). Range of movements was assessed at third month for one year after the fixator removal. Epiphyseal index was calculated by dividing the epiphyseal height at the middle point by the epiphyseal width at its widest points (normal range for children was 0.8-0.9) [13]. The percentage of the femoral head at the widest transverse diameter that protrudes laterally to Perkin's vertical line is defined as “uncoverage percentage” [7]. The functional status of these patients was assessed by Harris Hip score.

## 3. Results

A total of 12 patients (8 males and 4 females) were included in this study. The mean age at onset of symptoms was 9.1 years (range 8.2–9.8 years) and the mean age at the time of presentation to us for the management was 9.8 years (range 8.9–10.5 years). Seven hips were on the right side and the remaining five were on the left side. In the present study, there was no case with bilateral hip involvement. The demographic characteristics of these patients and the management details are given in Table 1.

The eight hips were Herring C and the rest were at Herring B at the time of presentation. All female patients were of Herring C; early walking with fixator on was started at mean 1.8 days. The distraction was started at mean 3.1 days and was performed for mean 13.9 days (range 11–16 days). However, in our present work, no further distraction was made as the end point mentioned achieved (distraction was not released). In only one of our patients, Shenton's arch was maintained; otherwise, in the rest, distraction was stopped till distraction caused pain. The mean length of follow-up of these hips was 32.4 months (range 24.6–36.2 months). No additional surgical procedures were performed in any of these patients. The clinical outcomes were shown in Table 2.

**Figure 1:**
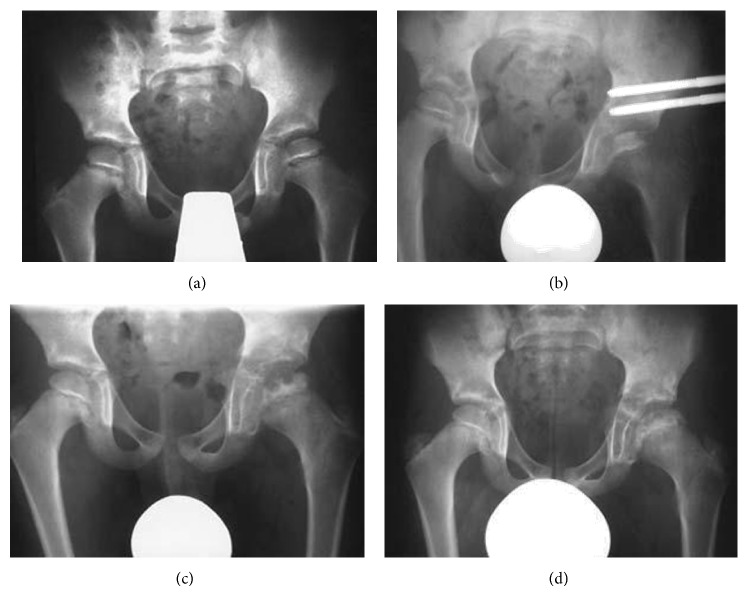
A 10.2-year-old female patient with Perthes' disease for the last eight months. (a) Managed with hinged monolateral external fixator, showing increased sclerosis of epiphysis with maintaining the epiphyseal height (b). At 1-year follow-up further collapse of epiphysis with osteolysis (c) was evident, which was resolved by last follow-up at 33.4 months with improved epiphyseal index (d).

**Figure 2:**
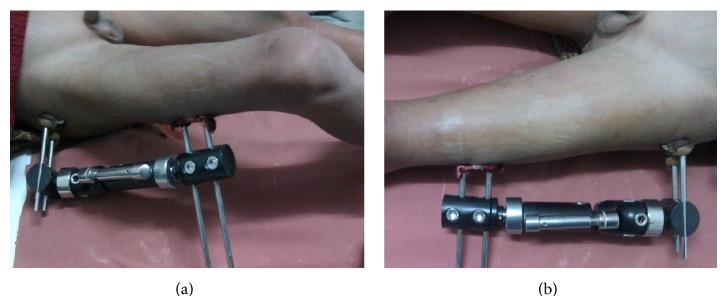
Clinical photographs of monolateral external fixator in place (a) with range of flexion movement at hip with fixator on (b).

The mean epiphyseal index measured at the end of 24 months was significantly improved from 0.69 to 0.75 (*P* = 0.005). It could not be established whether any epiphyseal collapse would have occurred if the arthrodiastasis had been avoided due to short follow-up. During the follow-up, the change in the percentage of uncovered head was insignificant in this study. The appearance of osteolysis around the dead bone was reduced in all patients. During the treatment, four patients developed the pin tract infection, which was controlled by local dressing and antibiotics. No case of implant failure or stress fracture was seen. At the last follow-up, Harris Hip score improvement was observed from 56 to 86, which was significant. As yet, it is too early for all the patients to be given a grading according to Stulberg et al. [14], as that can be done only at skeletal maturity (Figures 1 and 2).

## 4. Discussion

The present longitudinal prospective study was conducted with the aim of evaluating the effect of hip distraction in cases of Perthes' disease in elderly children (more than 8 years). Our research hypothesis was that hip arthrodiastasis would improve the outcome of Perthes' disease in the selected group of patients with risk of poor outcome by halting the further defragmentation without altering the anatomy of the region. This modality of treatment is relatively new treatment for Perthes' disease. No conventional surgical modality of treatment has shown definite and significant improvement of outcome or any change in the course of the disease [15]. This is partly due to the difficulty in evaluating the effect of the treatment on a disease that has a variable course, duration, and outcome and also because of methodological difficulties such as lack of a control group [7]. The possible advantage of this modality is that it relieves pressure on the necrosed portion of the neck, without altering the anatomy of the region. It was observed that the maximum collapse occurred within the first seven months of onset of symptoms [16], so we only included children with symptoms for less than 1 year. Joseph et al. [17] managed the adolescent onset Perthes' disease and recommended that the treatment should be started early, as the potential to regain epiphyseal height by remodeling is limited in the older group. Several studies have been performed to evaluate the role of arthrodiastasis in Perthes' disease. Kocaoglu et al. [18] applied the joint distraction with an Ilizarov fixator in 11 patients with mean age of 7.5 years. Due to fixator at hip region, patients' were not able to perform any movements. Further, it was noted that the disease process progressed more rapidly in the healing phase. However, because of the high incidence of the complications, they did not recommend this technique for routine use. Guamiero et al. [19] studied 36 children with Perthes' disease, in which eighteen were treated with femoral varus osteotomy and the rest by arthrodiastasis of hip by an external fixator. They observed satisfactory results in both groups, but the remodeling occurred faster in cases treated by arthrodiastasis. Moreover, Maxwell et al. [7] carried out a prospective trial in boys over the age of 8 years and girls over 7 years at the time of the onset of symptoms and found this modality as an effective method in preventing further femoral collapse. Amer and Khanfour [3] treated 30 patients by hip distraction using minimal soft tissue release and a simple Ilizarov construct. After an average follow-up of 3.6 years, they observed an improvement in the range of movements, pain, and superior and lateral subluxation of head, with a statistically significant difference between pre- and postoperative values. Hosny et al. [20] treated 29 children (older than 8 years at onset) with Perthes' disease (lateral pillar type C or B). They observed improvement in range of movements of hip in about 93% of patients. They concluded that hip distraction without soft tissue release seems to be a valid treatment option in cases with Perthes' disease compared to the conventional treatment. Laklouk and Hosny [21] managed 53 patients with Perthes' disease with a combination of soft tissue release and joint distraction with external fixator. Out of these, 32 were treated by hinged monolateral external fixator and the rest by Ilizarov external fixator. Further, they evaluated 19 of these patients, who attained skeletal maturity, and observed that this method not only improves range of movements but also reduces the superior and lateral subluxation and provides a better radiographic sphericity of the femoral head. Moreover, they recommended that this method can be used when other methods of treatment are contraindicated. Our present study showed improvement in the range of movements by arthrodiastasis of the hip and it was found to inhibit the further epiphyseal collapse in the treated patients.

Noonan et al. [8] retrospectively reviewed the effectiveness of femoral varus osteotomy in 18 hips of 17 children over the age of 9 years. In this study, they concluded that, for patients over the age of 10 years, a varus osteotomy did not influence the natural history of the disease. Hsu et al. [10] conducted a study to determine whether shelf arthroplasty for Perthes' disease (1) prevents the onset of early hip osteoarthritis; (2) improves pain, range of movement, activity, and functional outcome; (3) maintains or improves femoral head containment, sphericity, and congruency; (4) changes the acetabular index; (5) is associated with a low rate of complications. They reviewed medical literature from 1966 to 2009 using the search terms Perthes, shelf procedure, and acetabuloplasty and finally concluded that while radiographic measurements indicate improved coverage of the femoral head after shelf acetabuloplasty for Perthes' disease, available evidence did not document the procedure that prevents early onset of osteoarthritis or improves long term function. Thompson [22] reviewed Salter osteotomy as a technique of surgical containment in Perthes' disease. They concluded that Salter osteotomy is an effective method of surgical treatment that can alter the natural history of Perthes' disease. In a retrospective review of 24 patients (mean age 9.8 years) with late onset Perthes' disease treated with shelf acetabuloplasty, Wright et al. [23] recommended that the procedure deserves further evaluation in any future randomized controlled trial of the management of late onset Perthes' disease. Hosalkar et al. [11] evaluated 20 triple innominate osteotomies for Perthes' disease. They concluded that it improved lateral head coverage in all cases; however, this study was conducted in children with mean age of 3.8 years. These traditional open surgical procedures not only have a complication, like infection, stiffness of the hip, shortening, and resurgery for implant removal, but also alter the local anatomy. In some studies [23, 24] these complications overweigh the benefits of intervention. The present study observes that arthrodiastasis of hip not only preserves the local anatomy but also maintains the local soft tissue integrity.

Maxwell et al. [7] observed that variable nature of Perthes' disease made the condition difficult to study. The use of different outcome measures led to further confusion. To date, no study has used extent of epiphyseal collapse in order to assess the early surgical procedures, so comparison of our results with other studies is not possible at present. As, to date, the search for the optimum treatment of late onset Perthes' is still going on, our early results with arthrodiastasis of hip in this condition show considerable potential of this technique, but the limitation of the present study is a short term follow-up. However, we hope that, with long term follow-up until the skeletal maturity, it may become apparent whether arthrodiastasis of hip gives a better long term result in older children with Perthes' disease.

## Figures and Tables

**Table 1:** Demographic characteristic of patients.

Case number	Gender	Age at onset of symptoms (years)	Preop Herring grades	Age at fixator application (years)	Duration of distraction (d)	Herring grade at final follow-up	Length of follow-up (months)
1	M	8.2	C	8.9	11	C	61.2
2	M	9.1	B	9.9	13	B	53.9
3	F	9.4	C	10.2	11	C	58.4
4	M	8.6	B	9.5	15	B	60.7
5	M	8.9	C	9.6	14	C	54.9
6	M	9.8	B	10.5	16	C	55.4
7	M	9.6	B	9.8	13	C	52.9
8	F	9.3	C	9.7	15	C	56.1
9	F	8.7	C	9.3	12	C	60.7
10	M	8.9	C	9.7	16	C	59.9
11	M	9.2	C	10.2	16	C	61.1
12	F	9.3	C	10.4	15	C	49.6

**Table 2:** Clinical outcomes.

Parameter	Preop	Last follow-up
Trendelenburg sign	**+**ve	**+**ve
Limb length discrepancy (av. cm)	2.3	2.1
Range of motion (mean degree)		
Flexion	31	78
Extension	−ve	−ve
Abduction	10	28
Adduction	21	26
Ext. rotation	21	30
Int. rotation	5	15
